# Decoding the regulatory role of ATP synthase inhibitory factor 1 (ATPIF1) in Wallerian degeneration and peripheral nerve regeneration

**DOI:** 10.1002/EXP.20230098

**Published:** 2024-03-19

**Authors:** Yun Qian, Zhiwen Yan, Tianbao Ye, Victor Shahin, Jia Jiang, Cunyi Fan

**Affiliations:** ^1^ Department of Orthopedics Shanghai Sixth People's Hospital Affiliated to Shanghai Jiao Tong University School of Medicine Shanghai People's Republic of China; ^2^ Shanghai Engineering Research Center for Orthopaedic Material Innovation and Tissue Regeneration Shanghai People's Republic of China; ^3^ Department of Cardiology Shanghai Sixth People's Hospital Affiliated to Shanghai Jiao Tong University School of Medicine Shanghai People's Republic of China; ^4^ Institute of Physiology II University of Münster Münster Germany

**Keywords:** ATP synthase inhibitory factor 1, CCR2/CCL2 signaling, macrophage infiltration, peripheral nerve injury, Wallerian degeneration

## Abstract

ATP synthase inhibitory factor 1 (ATPIF1), a key modulator of ATP synthase complex activity, has been implicated in various physiological and pathological processes. While its role is established in conditions such as hypoxia, ischemia‐reperfusion injury, apoptosis, and cancer, its involvement remains elusive in peripheral nerve regeneration. Leveraging ATPIF1 knockout transgenic mice, this study reveals that the absence of ATPIF1 impedes neural structural reconstruction, leading to delayed sensory and functional recovery. RNA‐sequencing unveils a significant attenuation in immune responses following peripheral nerve injury, which attributes to the CCR2/CCL2 signaling axis and results in decreased macrophage infiltration and activation. Importantly, macrophages, not Schwann cells, are identified as key contributors to the delayed Wallerian degeneration in ATPIF1 knockout mice, and affect the overall outcome of peripheral nerve regeneration. These results shed light on the translational potential of ATPIF1 for improving peripheral nerve regeneration.

## Graphical abstract

This study aims to understand ATP synthase inhibitory factor 1's (ATPIF1's) role in peripheral nerve regeneration (PNR) using a murine model of sciatic nerve crush injury. Histological exams and behavior assessments revealed ATPIF1 absence hindered nerve tissue reconstruction, delaying sensory and functional recovery. RNA‐sequencing showed reduced immune responses in ATPIF1 knockout mice, particularly CCR2/CCL2 signaling‐mediated macrophage infiltration, slowing Wallerian degeneration and impeding axonal regeneration.

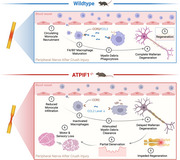

## 1 INTRODUCTION

Peripheral nerve injury (PNI) is a devastating condition that affects millions of people worldwide.^[^ ^1^ ^]^ In a study conducted by Noble et al., PNI was reported in 2.8% of patients among a total of 5,777 individuals who experienced traumatic injuries.^[^ ^2^ ^]^ PNI can result from various causes, such as trauma, tumorectomy, or diabetes. It usually leads to sensory and motor impairments, chronic pain, and reduced quality of life.^[^ ^3^ ^]^ The clinical management of PNI predominantly relies on direct end‐to‐end epineural suturing. However, in cases of nerve defects where tension‐free neurorrhaphy is unattainable, the application of nerve grafts (such as autografts, xenografts, and allografts) or artificial nerve conduits becomes necessary for nerve repair.^[^ ^4^ ^]^

The peripheral nerve is among the few tissues that can regenerate spontaneously in human body. However, the peripheral nerve regeneration (PNR) process is often slow, incomplete, and influenced by multiple factors.^[^ ^5^ ^]^ One of the key events that occurs after PNI is Wallerian degeneration, which is the rapid and orderly breakdown of the distal axon and its myelin sheath.^[^ ^6^ ^]^ Wallerian degeneration is essential for PNR, as it clears the debris and releases growth factors that promote axonal sprouting and Schwann cells (SCs) proliferation^[^ ^7^ ^]^.

ATP synthase inhibitory factor 1 (ATPIF1) is a key regulator of the ATP synthase complex, which is responsible for producing ATP.^[^ ^8^ ^]^ ATPIF1 has been implicated in various biomedical processes, for instance visual impairment, ischemia‐reperfusion injury, apoptosis, and carcinoma.^[^ ^9^ ^]^ However, the role of ATPIF1 in PNR and Wallerian degeneration remains elusive.

**FIGURE 1:**
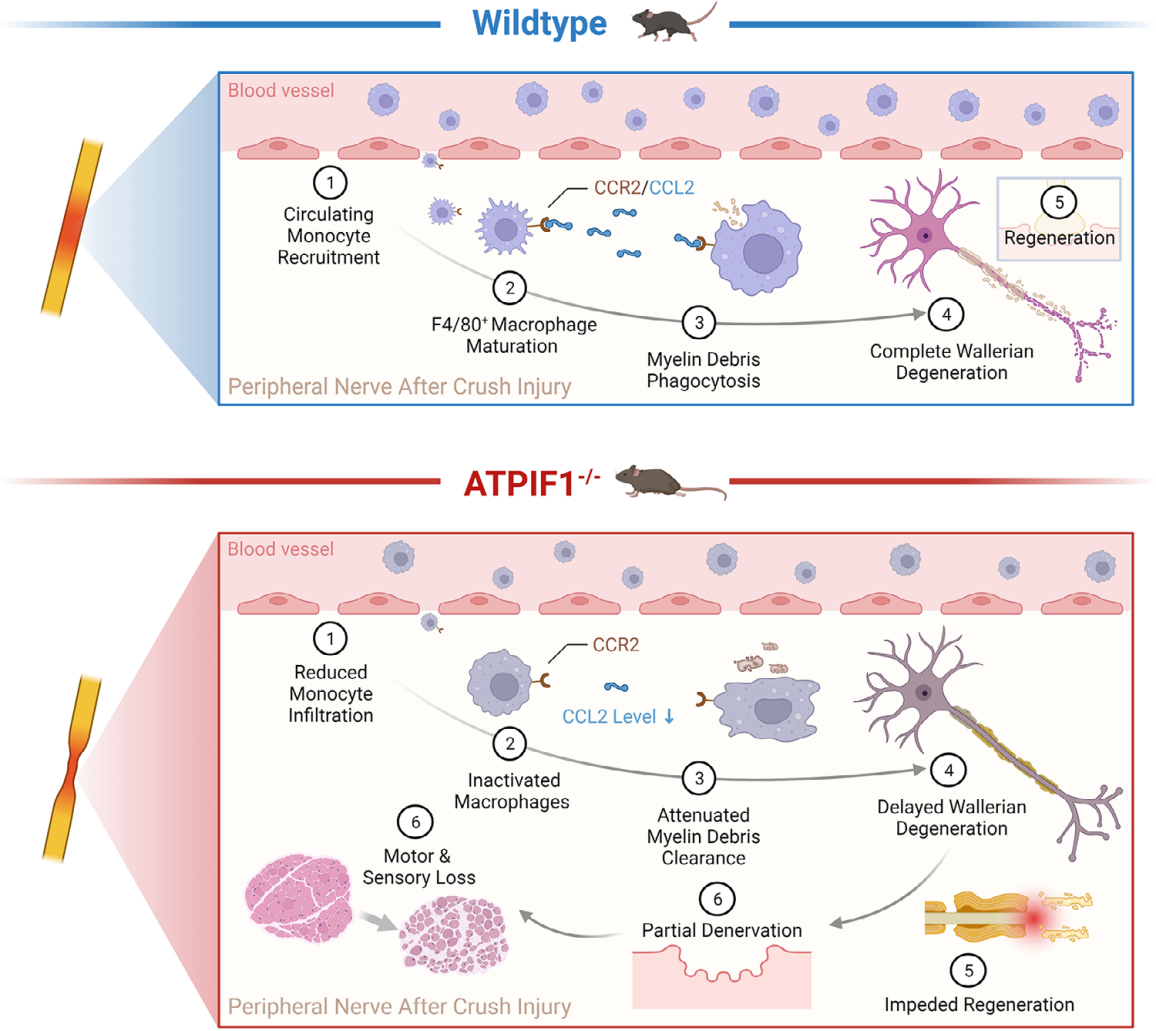
Schematic representation of key findings. In WT strain receiving nerve crush surgery, the circulating monocytes are recruited to the injury site through the CCR2/CCL2 signaling axis, leading to the maturation of F4/80‐positive macrophages. A collaborative effort between macrophages and SCs ensues, facilitating the effective clearance of myelin and axon debris distal to the crush site, a process recognized as Wallerian degeneration. Following complete Wallerian degeneration, injured sciatic nerves undergo regeneration across the injury site, ultimately reinnervating the target organ. In IF1KO mice, a notable reduction in monocyte infiltration post‐PNI is observed, accompanied by decreased expression of CCR2/CCL2. This diminishes the quantity and activities of macrophages within the injury site, resulting in inadequate clearance of myelin and axon debris, indicative of incomplete Wallerian degeneration. The accumulation of surplus myelin and axon debris forms a physical barrier, impeding axonal regeneration. Consequently, this incomplete Wallerian degeneration leads to partial denervation of the target muscle and skin, culminating in severe motor and sensory loss. This figure was created with BioRender.com.

This investigation aims to elucidate the involvement of ATPIF1 in PNR with the murine model of sciatic nerve crush injury. Comprehensive histological examinations and behavioral assessments were conducted to juxtapose PNR outcomes between wild‐type (WT) and ATPIF1 knockout (IF1KO) strain. Laboratory observations revealed the absence of ATPIF1 hindered structural reconstruction of peripheral nerve tissues, leading to a delayed recovery in both sensory and functional aspects post‐injury. To delve into the potential mechanisms underpinning the impact of ATPIF1 on PNR, the results from RNA‐sequencing unveiled a significant attenuation in immune responses within IF1KO mice. Notably, the CCR2/CCL2 signaling‐mediated macrophage infiltration emerged as a pivotal factor in this phenomenon. The reduction in macrophage quantity and activities contributed to a delay in completing Wallerian degeneration, allowing axon and myelin debris to impede axonal regeneration (Figure 1). This study highlights ATPIF1 as a translational molecular target for treating PNI.

## 2 RESULTS

### 2.1 The knockout of ATPIF1 impeded PNR in a rodent sciatic nerve crush model

**FIGURE 2:**
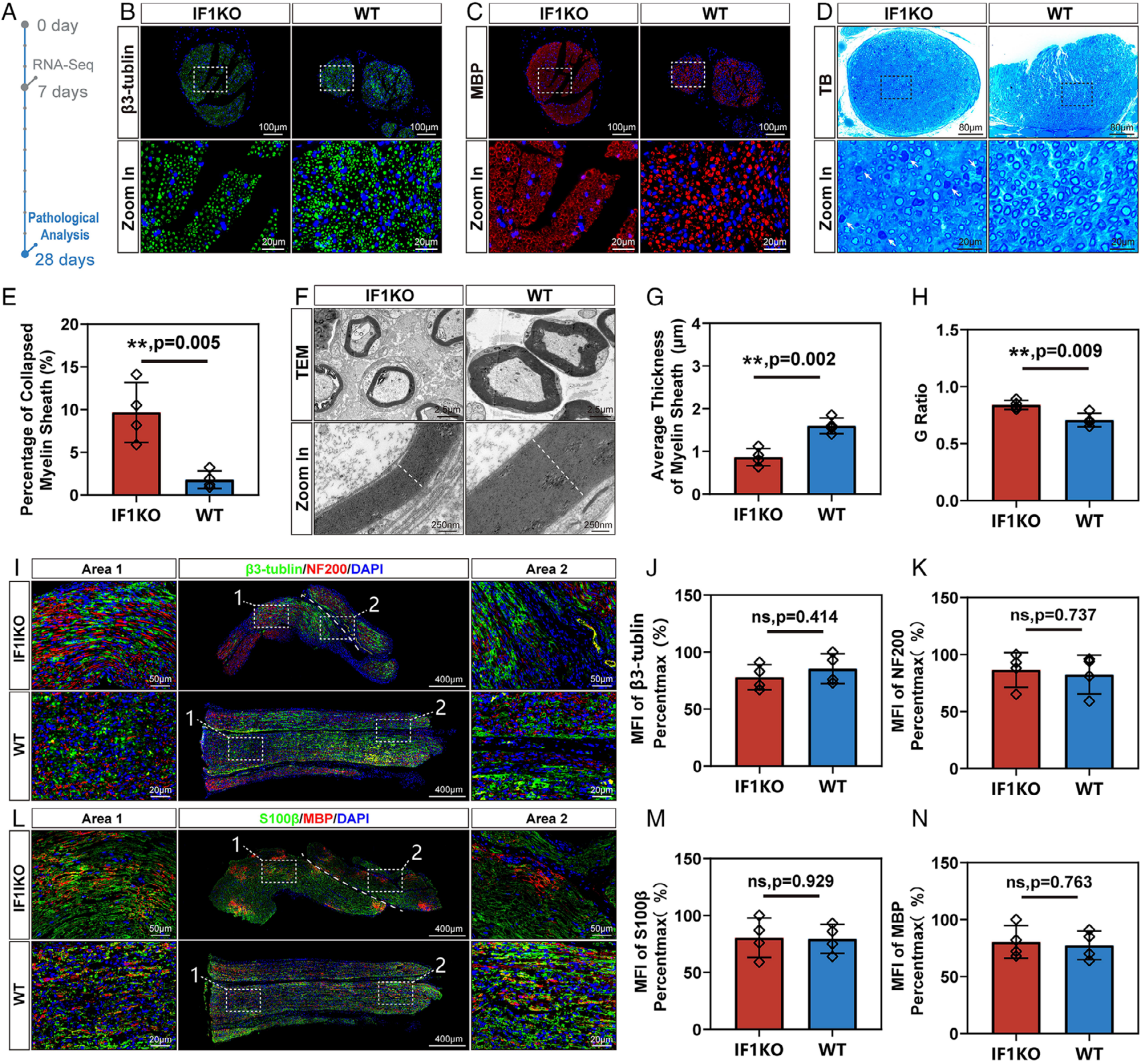
Pathological analysis of sciatic nerves 28 DPI in IF1KO and WT mice. (A) Illustration detailing the experimental timeline. (B) Transverse section showing immunofluorescent staining of crushed sciatic nerves (Green: β3‐tubulin, Blue: DAPI). (C) Transverse section displaying immunofluorescent staining of crushed sciatic nerves (Red: MBP, Blue: DAPI). (D) Transverse section presenting toluidine blue (TB) staining of crushed sciatic nerves. White arrows indicate collapsed myelin sheath. (E) Statistical analysis of the percentage of collapsed myelin sheath. (F) TEM images of crushed sciatic nerves. (G) Statistical analysis of the average thickness of the myelin sheath. (H) Statistical analysis of the G ratio. (I) Longitudinal section displaying immunofluorescent staining of crushed sciatic nerves (Green: β3‐tubulin, Red: NF200, Blue: DAPI). White dashed line indicates the discontinuous area. (J) Statistical analysis of the mean fluorescent intensity (MFI) of β3‐tubulin. (K) Statistical analysis of the MFI of NF200. (L) Longitudinal section exhibiting immunofluorescent staining of crushed sciatic nerves (Green: S100β, Red: MBP, Blue: DAPI). White dashed line indicates the discontinuous area. (m) Statistical analysis of the MFI of S100β. (N) Statistical analysis of the MFI of MBP. Statistical analysis for (E, G, H, J, K, M, N) was performed using unpaired two‐tailed Student's *t*‐test (*n* = 4). The data were normalized relative to the maximum reading within the entire dataset in (J, K, M, N).

To assess the impact of ATPIF1 on PNR, we employed a classical sciatic nerve crush model in mice, and collected nerve tissues distal to the crush site 28 days post‐injury (DPI) (Figure 2A). Transverse slices were immunofluorescent stained with axonal marker β3‐tublin and myelin marker myelin basic protein (MBP) (Figure 2B,C). Additionally, toluidine blue staining of semi‐thin sections was performed to visualize the axon‐myelin unit (Figure 2D).

In the IF1KO mice, a heightened likelihood of myelin sheath collapse was observed, indicative of compromised neural regeneration (Figure 2E). This finding was consistent with transmission electron microscope (TEM) analysis (Figure 2F), illustrating a notable decrease in myelin sheath thickness compared to the corresponding post‐injury time point in WT mice. (Figure 2G). The G ratio, a metric reflecting axon‐myelin structure maturation and reconstruction, was notably higher in the IF1KO group, underscoring inferior neural regeneration capacity (Figure 2H).

To validate these results, immunofluorescent staining of longitudinal sections of injured nerves was performed to evaluate additional markers, including NF200 (axon) and S100β (SCs). Although statistical significance wasn't observed in these markers (β3‐tubulin, NF200, MBP, and S100β), discontinuity in nerve fibers was evident (Figure 2I–N). This suggests that axons in IF1KO mice failed to traverse the injury site and regenerate distally. Cumulatively, these experiments confirm the detrimental impact of ATPIF1 knockout on PNR.

### 2.2 The knockout of ATPIF1 delayed the motor and sensory recovery after PNI

**FIGURE 3:**
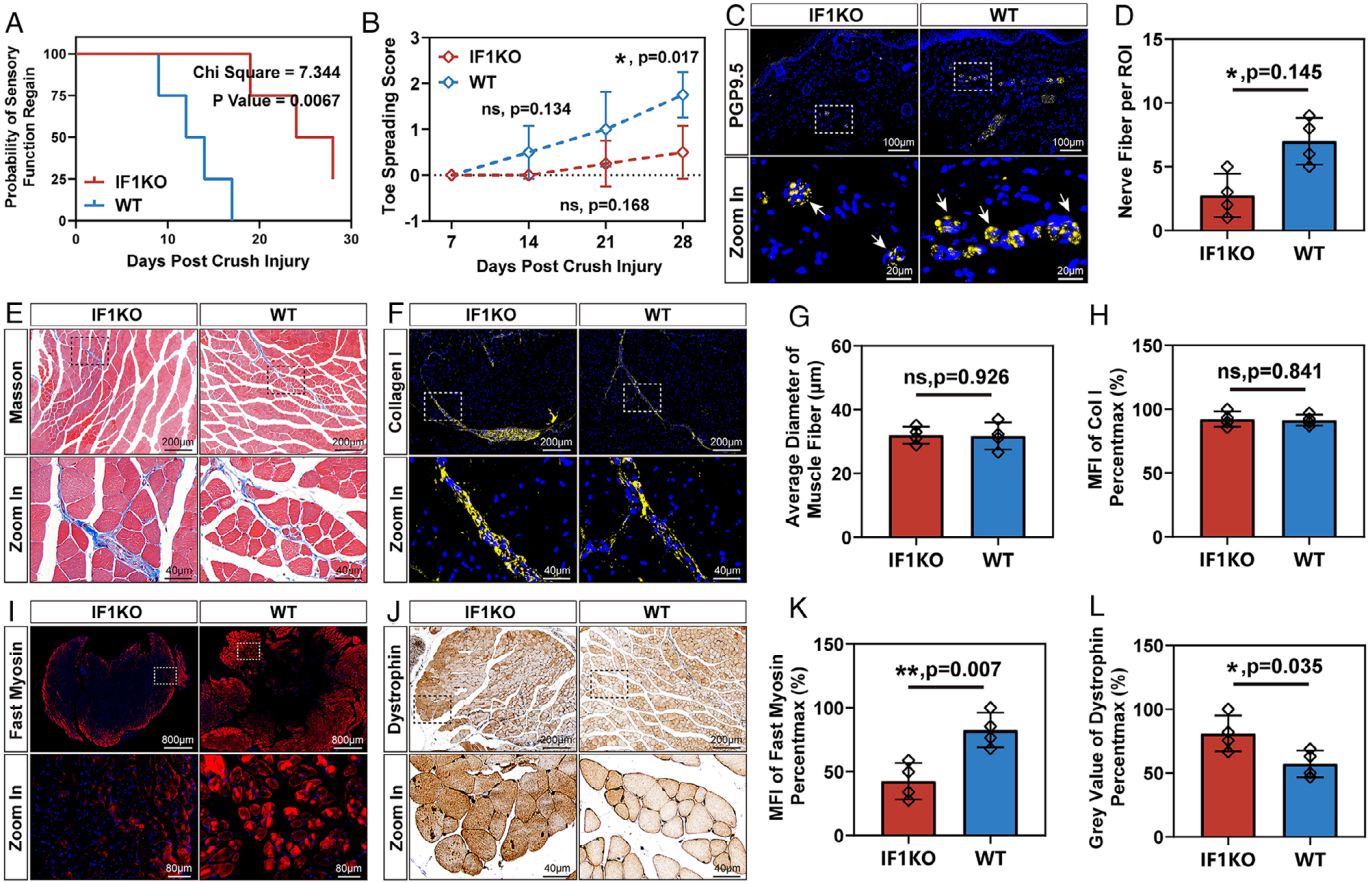
Motor and sensory function analysis 28 days post sciatic nerve crush injury in IF1KO and WT mice. (A) Measurement of sensory function restoration assessed by the reappearance of paw withdrawal response in the pinprick test. Statistical analysis was conducted using the Log‐rank (Mantel‐Cox) test. (B) Evaluation of toe spreading score at 7, 14, 21, and 28 DPI. (C) Immunofluorescent staining of hindpaw interdigital skin 28 DPI (Gold: PGP9.5, Blue: DAPI). White arrows indicate nerve fibers innervating the skin. (D) Quantitative analysis of nerve fibers per region of interest (ROI). (E) Masson’s trichrome staining of the gastrocnemius muscle 28 DPI. (F) Immunofluorescent staining of gastrocnemius muscle 28 DPI (Gold: Collagen I, Blue: DAPI). (G) Quantitative analysis of the average diameter of muscle fibers. (H) Quantitative analysis of MFI of collagen I. (I) Immunofluorescent staining of gastrocnemius muscle 28 DPI (red: fast myosin, blue: DAPI). (J) Dystrophin immunochemistry staining of gastrocnemius muscle 28 days after injury. (K) Quantitative analysis of MFI of fast myosin. (I) Semi‐quantitative analysis of the grey value of dystrophin. Statistical analysis for (B, D, G, H, K, L) was performed using unpaired two‐tailed Student's *t*‐test (*n* = 4). The data were normalized relative to the maximum reading within the entire dataset in (H, K, L).

The absence of ATPIF1 was found to intricately impact the timeline of motor and sensory recovery following PNI. Initiating with the pinprick test to evaluate sensory function, we recorded the day mice displayed a noticeable paw withdrawal in response to the pain‐triggering pinprick. Survival curve visualization revealed a median of 26 days for the IF1KO group, notably delayed compared to the 13 days median for the WT group. The Log‐rank (Mantel‐Cox) test affirmed a significant difference (*p* = 0.0067) in sensory recovery between these two strains (Figure 3A). Assessment of motor function recovery included observing the toe‐spreading reflex when mice were lifted by the tail. This reflex, governed by hind paw motor nerve regeneration, tends to fully spread the toes in healthy mice. Both strains exhibited a lack of this reflex 7 DPI, with the WT strain showing a superior probability of recovery at 14 and 21 days. A statistically significant difference (*p* = 0.017) emerged 28 DPI, mirroring the trend observed in sensory recovery (Figure 3B). Notably, motor recovery lagged behind sensory recovery after PNI.

Upon sacrificing the mice 28 DPI, we examined interdigital skin innervation using PGP9.5 immunofluorescent staining (Figure 3C). IF1KO mice displayed reduced nerve fibers within the skin, aligning with behavioral experiment results (Figure 3D).

Analysis of gastrocnemius muscle morphological changes employed Masson's trichrome and collagen I staining (Figure 3E,F). Calculations of average muscle fiber diameter and collagen I mean fluorescent intensity (MFI) were conducted to evaluate muscle atrophy and fibrosis status, indicative of PNI functional outcomes (Figure 3G,H). Nevertheless, no statistically significant distinctions were detected, potentially attributed to the severity of the nerve crush injury not being sufficient to induce noticeable muscle atrophy and fibrosis. At the molecular level, diminished fast myosin expression and slightly elevated dystrophin protein expression in IF1KO mice indicated delayed muscle reinnervation (Figure 3I–L), providing insights into the intricate mechanisms underlying the observed functional outcomes.

### 2.3 The knockout of ATPIF1 delayed the Wallerian degeneration by attenuating the CCR2/CCL2 signal mediated inflammatory response after PNI

**FIGURE 4:**
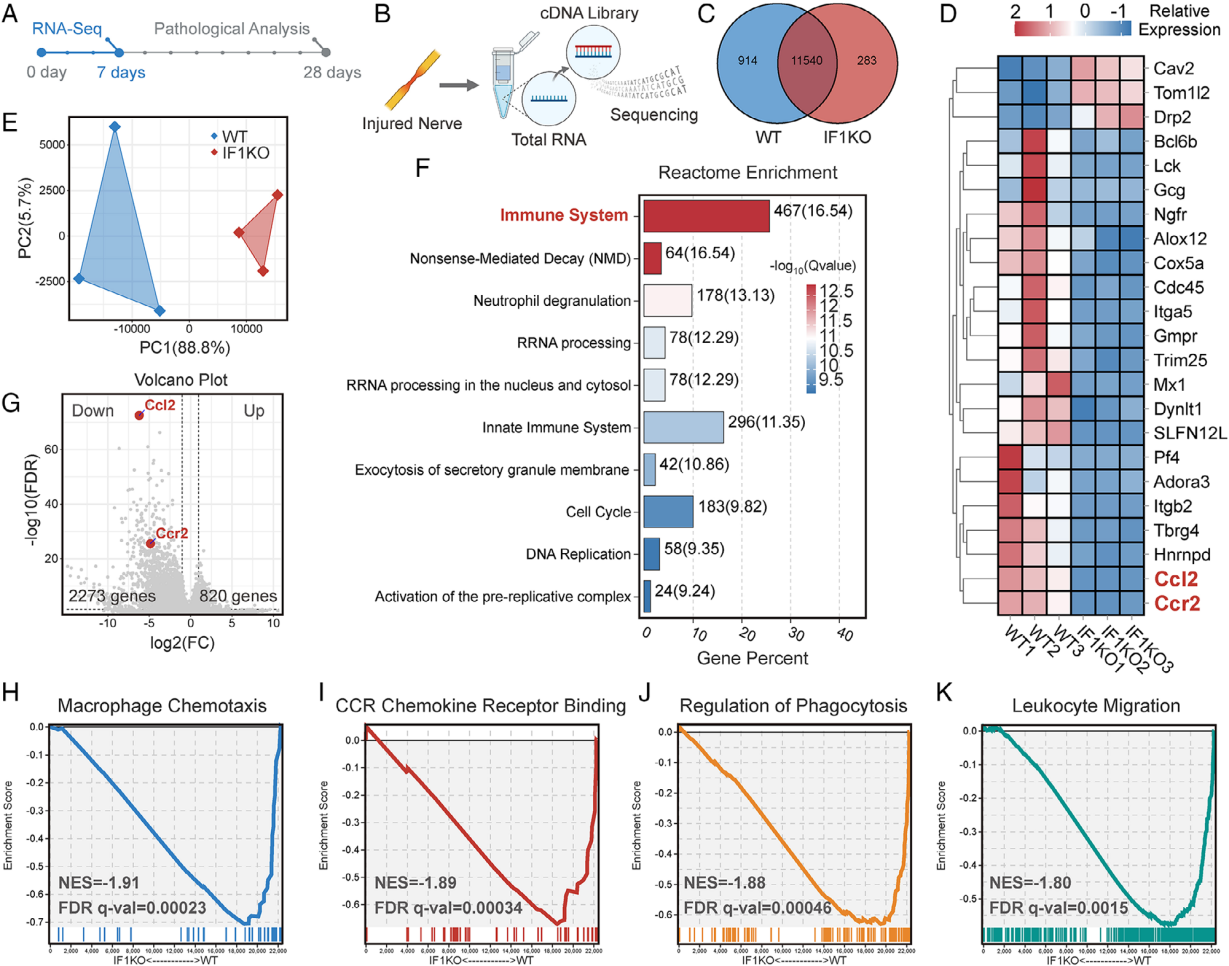
RNA sequencing of injured sciatic nerves in IF1KO and WT mice 7 DPI. (A) Overview of the experimental schedule. (B) Visual representation of the sciatic nerve RNA sequencing process (*n* = 3). (C) Venn diagram illustrating the overlapping RNA expression profile between the IF1KO and WT groups. (D) Heatmap depicting the relative expression levels of selected DEGs. (E) PCA plot demonstrating sample quality control. (F) Visualization of Reactome enrichment of the DEGs. (G) Volcano plot showing the DEGs. (H) GSEA analysis of the GO nomination “macrophage chemotaxis”. (I) GSEA analysis of the GO nomination “CCR chemokine receptor binding”. (J) GSEA analysis of the GO nomination “regulation of phagocytosis”. (K) GSEA analysis of the GO nomination “leukocyte migration”. The bioinformatics analysis threshold for DEGs was set at FDR < 0.05, fold change (FC) > 2, and *p* < 0.05. NES: Normalized enrichment score. FDR q‐val: False discovery rate q value.

To elucidate the molecular mechanisms behind the delayed neural regeneration in IF1KO mice, we collected injured nerve tissues 7 DPI and subjected it to RNA‐sequencing (Figure 4A,B). In accordance with the work by Shlomo Rotshenker,^[^ ^10^ ^]^ Wallerian degeneration is believed to initiate 2–3 DPI and reaches its peak around 7 DPI. Consequently, in this investigation, we selected 7 DPI as the designated timepoint to study Wallerian degeneration. The genome profiles of these two groups exhibited a considerable overlap, with 11,540 genes expressed in common. Notably, 914 genes exhibited unique expression in the WT group, while 283 genes were uniquely expressed in the IF1KO group. (Figure 4C). Genes such as Cav2, Tom1l2, and Drp2 showed higher expression levels in IF1KO mice, whereas Hnrnpd, Tbrg4, and Itgb2 exhibited higher expression levels in WT mice. Importantly, the IF1KO strain showed a significant decrease in the expression levels of CCR2 and CCL2 (Figure 4D). Sample quality was assessed using principal component analysis (PCA), revealing small differences within groups and a significant difference between the WT and IF1KO groups (Figure 4E). This affirmed the success of sample preparation. To further validate the accuracy of the RNA‐sequencing data, Pearson's correlation analysis was performed on each sample (Figure S1). The Pearson's correlation coefficients between IF1KO groups were 0.97927, 0.9673, and 0.98671, respectively. Conversely, the Pearson's correlation coefficients between WT groups were 0.92098, 0.88658, and 0.82676, respectively. These results provide additional confirmation of the sample quality and ensure the accuracy of the RNA‐sequencing. Differentially expressed genes (DEGs) were filtered using stringent criteria (FDR < 0.05, FC > 2, *p* < 0.05), resulting in 2,273 down‐regulated DEGs and 820 up‐regulated DEGs. Reactome enrichment analysis highlighted “immune system” as the most significantly enriched pathway (Figure 4F). Furthermore, the Kyoto Encyclopedia of Genes and Genomes (KEGG) analysis was conducted on the DEGs. Notably, the annotation “Immune System” encompassing 388 genes, secured the foremost position within the “Organismal Systems” category (Figure S2). As the CCR2/CCL2 signaling axis is crucial for monocyte infiltration after PNI, we visualized the expression of CCR2 and CCL2 on a volcano plot, revealing significant down‐regulation in the IF1KO strain (Figure 4G). The log_2_FC of CCL2 (WT vs IF1KO) was 6.174, with both *p*‐value and FDR < 0.0001. Similarly, the log_2_FC of CCR2 (WT vs IF1KO) was 4.813, with both *p*‐value and FDR < 0.0001. This led us to propose that the CCR2/CCL2‐mediated inflammatory response in IF1KO mice, triggered by PNI, was significantly attenuated, thereby delaying Wallerian degeneration.

To examine this hypothesis, we performed gene set enrichment analysis (GSEA) on the DEGs, focusing on gene ontology (GO) terms associated with this hypothesis. Annotations such as “macrophage chemotaxis” (GO:0048246, 42 genes) (Figure 4H), “CCR chemokine receptor binding” (GO:0048020, 56 genes) (Figure 4I), “regulation of phagocytosis” (GO:0050764, 109 genes) (Figure 4J), and “leukocyte migration” (GO:0050900, 361 genes) (Figure 4K) were all significantly down‐regulated in the IF1KO strain. The diminished “macrophage chemotaxis” substantiates the observation that IF1KO mice experienced reduced macrophage infiltration following PNI. The reduced “CCR chemokine receptor binding” reinforces the inference that the diminished inflammatory response was contingent on signals from the CCR chemokine receptor family. The attenuated “regulation of phagocytosis” buttresses the proposition that the IF1KO strain exhibited decreased and delayed Wallerian degeneration. The lowered “leukocyte migration” additionally bolsters the proposition that IF1KO mice induced less monocyte infiltration into the injury site after PNI.

**FIGURE 5:**
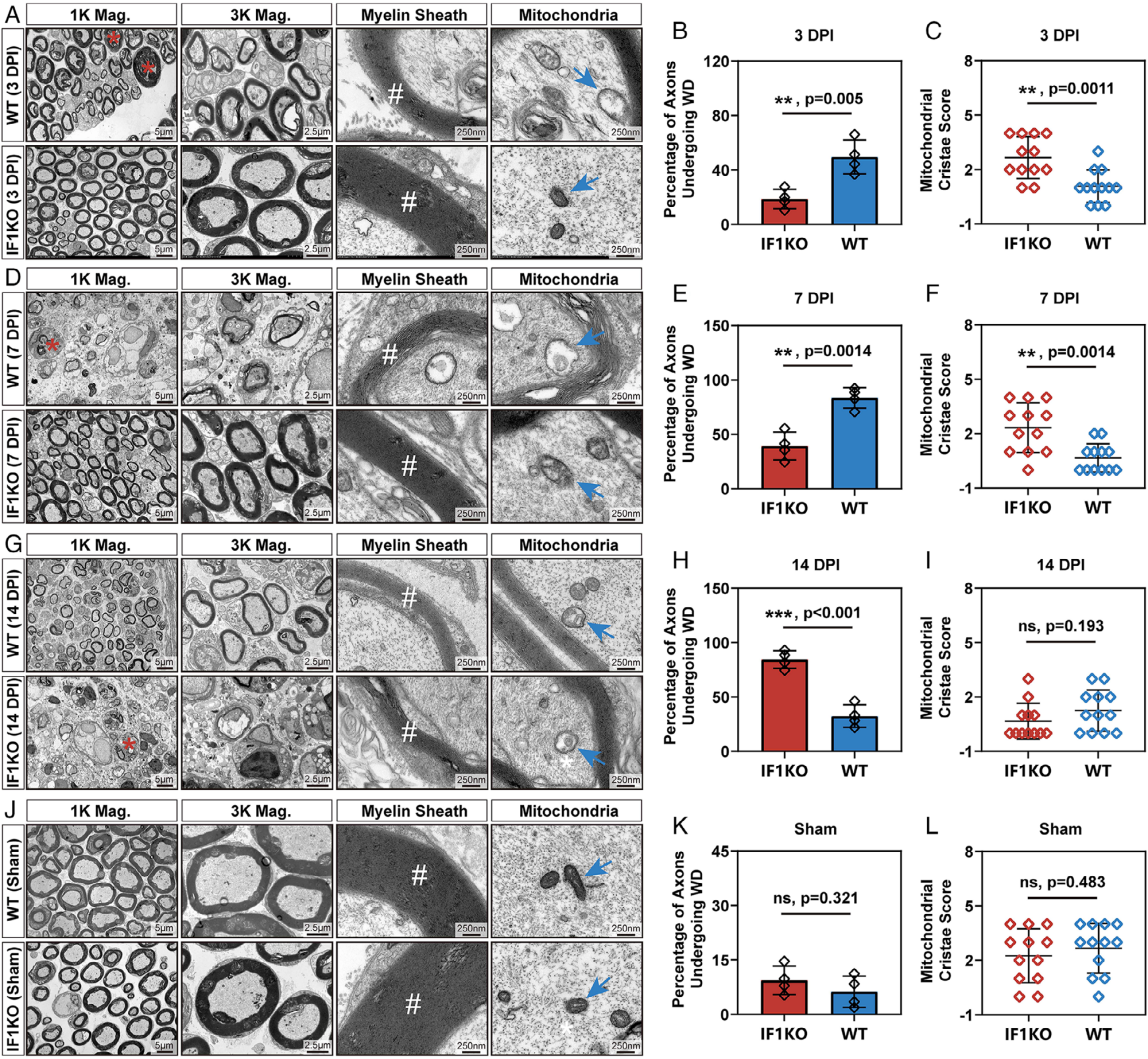
Validation of the delayed Wallerian degeneration in IF1KO mice. (A, D, G, J) TEM images at various magnifications (Mag.) illustrating the ultrastructure of sciatic nerves at 3, 7, and 14 DPI and in the sham group. Red asterisks denote axons undergoing Wallerian degeneration, white hashtags indicate the myelin sheath structure, and blue arrows mark the mitochondria. (B, E, H, K) Statistical analysis of the percentage of axons undergoing Wallerian degeneration at 3, 7, and 14 DPI and in the sham group. (C, F, I, L) Statistical analysis of the mitochondrial cristae score at 3, 7, and 14 DPI and in the sham group. The statistical analysis for (B, C, E, F, H, I, K, L) was performed using an unpaired two‐tailed student's *t*‐test (*n* = 4). Of note, three reads from one biologically independent sample were included in the statistical analysis of mitochondria cristae score in (C, F, I, L). WD: Wallerian degeneration.

To further substantiate the proposed hypothesis that IF1KO mice exhibit delayed Wallerian degeneration after PNI, TEM was utilized for observing myelin and axon structures related to Wallerian degeneration. Nerve segments distal to the injury site were harvested at 3, 7, and 14 days post‐PNI. In the WT group, three DPI, the myelin sheath began collapsing, and the axon‐myelin structure showed signs of fragmentation, indicating the onset of Wallerian degeneration (Figure 5A,B). Concurrently, in the WT group, mitochondria started swelling, and the cristae structure began to disappear (Figure 5C). However, in the IF1KO group, cytoskeletal integrity, unswollen mitochondria, and axon continuity were preserved. This phenomenon persisted even at 7 DPI in IF1KO mice. In contrast, in the WT group, the myelin‐axon structure was scarce, and phagocytotic vesicles, a hallmark of Wallerian degeneration, were observed (Figure 5D–F). Fourteen DPI, these pathological features of Wallerian degeneration were observed in the IF1KO group, while in the WT group, newly regenerated myelin‐axon structures were evident (Figure 5G–I). The regenerated structures were characterized by small inner axon diameters and thin myelin sheaths. Overall, this experiment validated delayed Wallerian degeneration in the IF1KO mice strain.

Furthermore, to address concerns regarding potential influences of IF1KO on Wallerian degeneration and other aspects of neural development, a sham group was included. Results indicated that nerve ultrastructure in both IF1KO mice and WT mice remained unchanged, exhibiting mature and distinctive myelin‐axon structures without obvious demyelination and mitochondrial dysfunction (Figure 5J–L). Motor function assessments in both juvenile and adult IF1KO mice showed no impairment (adult IF1KO mice: Supplementary Video S1; juvenile IF1KO mice: Supplementary Video S2). After sciatic nerve crush surgery, motor function was significantly compromised in IF1KO mice (Supplementary Video S3).

### 2.4 Macrophages but not SCs were responsible for the delayed Wallerian degeneration by CCR2/CCL2 signaling axis

**FIGURE 6:**
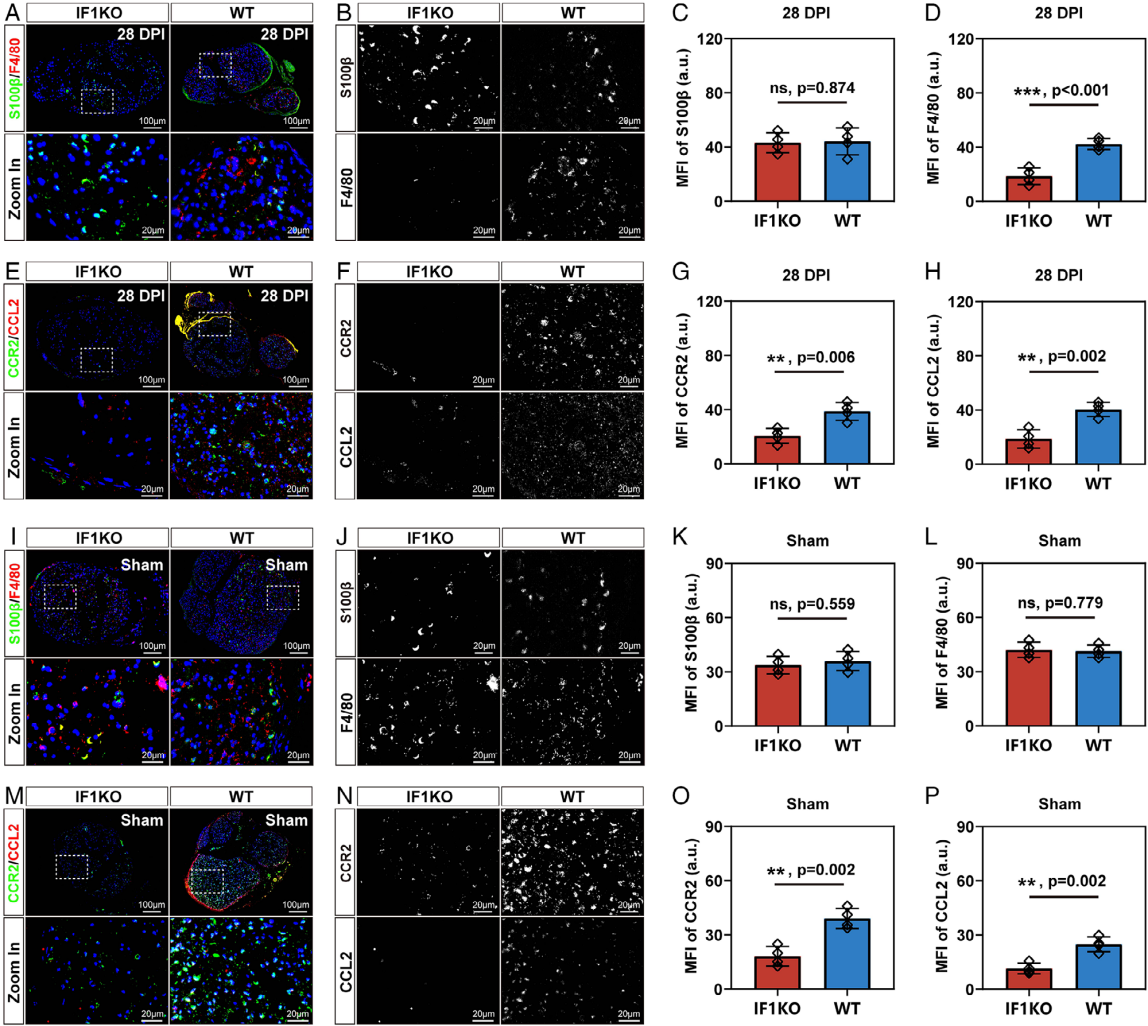
Validation of the proposed mechanism. (A) Transverse section showing staining of crushed sciatic nerves with S100β (green), F4/80 (red), and DAPI (blue). (B) Separate grey value image of S100β and F4/80 immunofluorescent staining. (C) Quantification of MFI for S100β (a.u. stands for arbitrary units). (D) Quantification of MFI for F4/80. (E) Transverse section displaying staining of crushed sciatic nerves with CCR2 (green), CCL2 (red), and DAPI (blue). (F) Separate grey value image of CCR2 and CCL2 immunofluorescent staining. (G) Quantification of MFI for CCR2. (H) Quantification of MFI for CCL2. (I) Transverse section showing staining of crushed sciatic nerves with S100β (green), F4/80 (red), and DAPI in sham group (blue). (J) Separate grey value image of S100β and F4/80 immunofluorescent staining. (K) Quantification of MFI for S100β. (L) Quantification of MFI for F4/80. (M) Transverse section displaying staining of crushed sciatic nerves with CCR2 (green), CCL2 (red), and DAPI (blue). (N) Separate grey value image of CCR2 and CCL2 immunofluorescent staining. (O) Quantification of MFI for CCR2. (P) Quantification of MFI for CCL2. The statistical analysis for (C, D, G, H, K, L, O, P) was performed using an unpaired two‐tailed student's *t*‐test (*n* = 4).

Wallerian degeneration involves the coordinated actions of two distinct cell types: macrophages and SCs. Nerve samples were collected 28 DPI, utilizing S100β as an SC marker and F4/80 as a macrophage marker for cell type detection within the injury site (Figure 6A,B). The levels of SCs between the WT and IF1KO groups did not exhibit significant differences (Figure 6C), while a notable decrease in macrophage levels was observed in the IF1KO group (Figure 6D). This outcome suggests that macrophages, not SCs, are accountable for the delayed Wallerian degeneration in IF1KO mice. To substantiate the role of the CCR2/CCL2 signaling axis in the diminished macrophage accumulation in the IF1KO strain, immunofluorescent staining and semi‐quantification of the MFI were conducted (Figure 6E,F). The results demonstrated a significant reduction in both CCR2 and CCL2 levels in the IF1KO group after PNI (Figure 6G,H). In order to address potential concerns regarding the impact of IF1KO on the developmental expression of the CCR2/CCL2 signaling axis, a sham group was included in our investigation (Figure 6I,J). Our results demonstrated that IF1KO did not exert discernible effects on the percentages of macrophages and SCs within the sciatic nerve tissue in the sham group (Figure 6K,L). Nevertheless, it was noteworthy that both CCR2 and CCL2 protein expressions were diminished in IF1KO mice, even under uninjured conditions (Figure 6M–P). Collectively, these findings support the model proposing that macrophages, rather than SCs, are responsible for the delayed Wallerian degeneration through the CCR2/CCL2 signaling axis.

## 3 DISCUSSION

Wallerian degeneration is a well‐characterized phenomenon that occurs after PNI, in which the distal axon and myelin sheath undergo rapid and orderly degradation.^[^ ^11^ ^]^ Wallerian degeneration is essential for PNR, as it clears the debris and creates a favorable environment for axonal sprouting and SCs proliferation.^[^ ^12^ ^]^ Nevertheless, the molecular and cellular mechanisms underlying Wallerian degeneration remain incompletely understood.

One of the key players in Wallerian degeneration is the macrophage, which is a type of immune cell that infiltrates the injured nerve and phagocytoses the axonal and myelin debris.^[^ ^13^ ^]^ Macrophages also secrete various factors that modulate the inflammatory response, the SCs phenotype, and the axonal growth.^[^ ^14^ ^]^ The role of macrophages in Wallerian degeneration has been extensively studied using various experimental approaches, such as pharmacological depletion, genetic manipulation, and transplantation.^[^ ^13^ ^]^

Previous studies have shown that macrophages are essential for Wallerian degeneration and PNR, as their depletion or deficiency leads to impaired clearance of debris, delayed or reduced expression of regeneration‐associated genes, and diminished functional recovery.^[^ ^13^ ^]^ Moreover, macrophages have been shown to exhibit different phenotypes and functions depending on the stage and location of Wallerian degeneration, for instance, the M1 or M2 polarization, or resident or recruited origin.^[^ ^15^ ^]^ These studies suggest that macrophages are dynamic and heterogeneous cells that exert diverse and complex effects on Wallerian degeneration and PNR. However, there are still many unresolved questions and challenges regarding the role of macrophages in Wallerian degeneration and PNR.

ATPIF1 is a mitochondrial protein that was first identified in 1998 as a factor that inhibits the ATP synthase complex, which is responsible for producing ATP from ADP and inorganic phosphate.^[^ ^16^ ^]^ ATPIF1 binds to the F1 subunit of the ATP synthase complex and reduces its activity, thereby affecting the mitochondrial membrane potential and cellular energy metabolism.^[^ ^17^ ^]^ The expression and function of ATPIF1 are regulated by various factors, such as oxygen levels, pH, calcium, and reactive oxygen species (ROS).^[^ ^18^ ^]^

ATPIF1 has demonstrated a protective function during hypoxia by maintaining mitochondrial membrane potential and preventing excessive ROS generation.^[^ ^19^ ^]^ In pathological cardiac hypertrophy, ATPIF1 upregulation triggers nonproductive tetramer formation in FoF1‐ATP synthase, impairing ATP synthase activity. This leads to mitochondrial ROS generation, stabilizing HIF‐1α and activating glycolysis. Cardiac‐specific ATPIF1 deletion in mice prevents this metabolic switch, offering protection against chronic stress‐induced remodeling.^[^ ^9a^ ^]^ Moreover, ATPIF1 has been shown to modulate apoptosis by interacting with pro‐apoptotic or anti‐apoptotic proteins, such as Bax, Bcl‐2, and Bcl‐xL.^[^ ^9c^ ^]^ Furthermore, ATPIF1 proved to be involved in cancer by regulating the glycolytic switch, the Warburg effect, the tumor microenvironment, and the chemoresistance.^[^ ^20^ ^]^

The role of ATPIF1 in neuronal diseases was also explored in animal models. Previous studies showed that ATPIF1 was upregulated in the brain after a stroke or traumatic brain injury and that ATPIF1 knockout or inhibition improved the neurological outcome and reduced the infarct size.^[^ ^21^ ^]^ Moreover, ATPIF1 is involved in neurodegenerative diseases. In Parkinson's disease (PD), mitochondrial dysfunction contributes to dopaminergic cell loss. ATPIF1 regulates mitochondrial energy metabolism. ATPIF1 treatment protected SH‐SY5Y cells against rotenone‐induced damage by maintaining ATP levels, inner membrane potential, and reducing oxidative stress. In PD mice, ATPIF1 administration improved motor performance.^[^ ^22^ ^]^ An independent study by Raffin et al. showed the role of ATPIF1 in human patients. Their findings indicated a positive correlation between baseline resting concentrations of plasma ATPIF1 and physical activity levels in older adults. Notably, this association was found to be partially mediated by apolipoprotein A‐I.^[^ ^23^ ^]^

In this investigation, we concentrated on examining the involvement of ATPIF1 in PNR through the utilization of a mouse model with sciatic nerve crush injury. We demonstrated that ATPIF1 knockout mice exhibited reduced PNR ability compared to wild‐type mice, as evidenced by impeded axonal growth and delayed functional recovery. We hypothesized that ATPIF1 might affect PNR by influencing the macrophage infiltration and activity in Wallerian degeneration in a CCR2/CCL2 dependent way by RNA‐sequencing.

CCL2, also recognized as monocyte chemoattractant protein‐1 (MCP‐1), was initially identified and purified from the culture supernatants of peripheral blood mononuclear cells and tumor cell lines in 1989.^[^ ^24^ ^]^ Being the first discovered and extensively studied CC family chemokine, CCL2 exhibits a preference for binding to its receptor, CCR2. Prior investigations have suggested that the CCL2/CCR2 signaling axis is implicated in various biological processes, including the promotion of pathological angiogenesis, facilitation of the survival and invasion of tumor cells, and the recruitment of immune inhibitory cells.^[^ ^24^ ^]^ In the peripheral nervous system, a preceding investigation^[^ ^25^ ^]^ elucidates the involvement of the CCL2/CCR2 axis in the dorsal root ganglia (DRG) concerning neurological pain. Moreover, this research underscores the therapeutic promise of modulating the CCL2/CCR2 axis in addressing chronic pain. Our research underscores the indispensability of CCL2/CCR2 signaling‐mediated macrophages infiltration for the completion of Wallerian degeneration, with ATPIF1 identified as an upstream regulator in this process. Our findings offer fresh perspectives on the molecular control of PNR and propose ATPIF1 as a promising therapeutic target for the treatment of PNI.

## 4 METHODS

### 4.1 IF1KO strain generation and animal surgery

All animal experiments were performed under the guideline of the Shanghai Jiao Tong University Institutional Animal Care and Use Committee (approval number: O_A2023071). The ATPIF1 knockout mouse model was established on a C57BL/6 background according to the previous publication.^[^ ^26^ ^]^ Homozygous KO mice (ATPIF1^−/−^) were obtained through the mating of heterozygous mice (ATPIF1^+/−^), and WT littermates (ATPIF1^+/+^) served as controls. This sciatic nerve crush surgery was performed according to the video guideline as published elsewhere.^[^ ^27^ ^]^

### 4.2 Immunofluorescent staining

At 28 DPI, sciatic nerve segments, gastrocnemius muscles, and hindpaw interdigital skin were collected from mice, along with a sham group. Samples were fixed in paraformaldehyde overnight, and nerve segments were cryosectioned into 10‐µm slices. Standard immunofluorescent staining protocols were applied, using primary antibodies: anti‐S100β, anti‐MBP, anti‐NF200, anti‐β3 tubulin, anti‐F4/80, anti‐CCR2, anti‐CCL2, anti‐Collagen I, anti‐Fast myosin, and anti‐PGP 9.5. After 2 h of secondary antibody staining and 10 min of DAPI counterstaining in the dark, samples were mounted with Fluoromount‐G and sealed. Fluorescent images were captured using a high‐resolution scanner (3D HISTECH) and analyzed in ImageJ, measuring fluorescence intensity by mean gray values within regions of interest.

### 4.3 Transmission electron microscope observation

The sciatic nerve segments collected at 3, 7, 14, and 28 DPI were processed following standard TEM procedures. Briefly, samples were fixed in 2.5% glutaraldehyde, post‐fixed in 1% osmium tetroxide, dehydrated, embedded in epoxy resin, and cut into ultrathin sections. After staining, samples were observed using TEM (JEOL JEM‐1400) at 80 kV. Image analysis was performed with ImageJ software to quantify ultrastructural features in the injured nerves. The mitochondrial cristae score, adapted from a prior publication,^[^ ^28^ ^]^ was evaluated on a scale of 0–4. Criteria for scoring included: 0—no sharply defined cristae, 1—over 50% of mitochondrial area lacking cristae, 2—over 25% lacking cristae, 3—numerous irregular cristae covering more than 75% of the area, and 4—numerous regular cristae.

### 4.4 Toluidine blue staining

The peripheral nerve segments were collected from mice 28 DPI and underwent a process similar to TEM sample preparation, with the exception that the nerve segments were cut into semi‐thin sections (1 µm). These sections were stained with a diluted Toluidine blue solution (0.1%) for 10 min, washed with water, covered with Permount, and coverslipped. High‐resolution images were obtained using a 3D HISTECH scanner.

### 4.5 Masson trichrome staining for gastrocnemius muscle

At 28 DPI, gastrocnemius muscles were collected from mice, fixed in 10% neutral buffered formalin, and processed for paraffin embedding. Tissue sections (5 µm) were stained with Masson trichrome solution to distinguish muscle fibers (red), collagen (blue), and nuclei (black). High‐resolution images were acquired using a high‐resolution scanner (3D HISTECH), and morphometric analysis was performed using ImageJ software to quantify relevant parameters in the regions of interest.

### 4.6 Immunohistochemistry staining for dystrophin protein in gastrocnemius muscle

Gastrocnemius muscles collected at 28 DPI were fixed, embedded, and sectioned for analysis. Sections were subjected to immunohistochemistry staining with anti‐dystrophin antibody (1:200, abcam), followed by secondary antibody treatment. The images were acquired using a high‐resolution scanner (3D HISTECH). Dystrophin expression was quantified using ImageJ software by measuring mean optical density in regions of interest.

### 4.7 Behavior analysis

Sensory recovery was assessed using a pinprick assay^[^ ^29^ ^]^ on mice habituated on wire mesh cages. After a 30‐min habituation, the paw's plantar surface was gently touched with an insect pin, and a positive response was recorded if the animal withdrew its paw quickly. A blinded observer conducted the scoring, and the saphenous territory served as a positive control.

To assess motor recovery after nerve injury, the toe spreading reflex was measured according to a previous publication.^[^ ^29^ ^]^ An independent study showed that the toe spreading reflex is more sensitive to test the locomotor function recovery than catwalk gait analysis.^[^ ^30^ ^]^ Mice were assessed for the toe spreading reflex, with scores ranging from 0 to 2 based on the degree of spreading. A full score of 2 indicated complete, wide, and sustained toe spreading for at least 2 s. Evaluations were conducted twice per session, with a 45‐minute interval. The scoring was done by a blinded observer.

### 4.8 Transcriptome analysis

Total RNA was isolated from the sciatic nerve tissues of IF1KO mice at 7 DPI, constituting the experimental group, while RNA from the WT group at the same post‐injury time point served as the control. After quality checks, these samples underwent RNA‐seq at GENE DENOVO, China. Upon verifying sample quality, transcript profiles of the experimental and control groups were meticulously compared for DEG identification using DESeq2 (version 1.24.0) analysis. Significance was set at *p* < 0.05, with a fold change cutoff of 2 and FDR < 0.05. Additional analyses, including GO term, KEGG analysis and Reactome analyses with a *p*‐value cutoff < 0.05, were conducted using the Omicsmart application. As specified in a prior publication,^[^ ^31^ ^]^ the genome version “Ensembl_release106” was utilized in this investigation. All RNA‐sequencing data and relevant bioinformatics results are provided in the Supplementary Table S1.

### 4.9 Statistics

GraphPad Prism software was utilized for statistical analysis, and the data are expressed as mean ± standard deviation. The specific statistical methods are detailed in the legends of the corresponding figures. Statistical significance was determined with a threshold of p < 0.05 (ns, not significant; *p < 0.05, **p < 0.01 and ***p < 0.001).

## AUTHOR CONTRIBUTIONS

Zhiwen Yan and Yun Qian conceptualized the study. Yun Qian, Zhiwen Yan, and Tianbao Ye performed the experiments. Cunyi Fan, Victor Shahin and Yun Qian reviewed the literature and commented on the manuscript. Yun Qian and Zhiwen Yan drafted the manuscript. Cunyi Fan, Jia Jiang, and Yun Qian revised the manuscript. All authors read and approved the final version.

## CONFLICT OF INTEREST STATEMENT

The authors declare no conflicts of interest.

## Supporting information

Supporting Information

Supporting Information

Supporting Information

Supporting Information

Supporting Information

## Data Availability

All data needed to evaluate the conclusions in the paper are present in the paper and the supporting information. Additional data related to this paper may be requested from the authors.
