# Supplementary material for: Decoding the regulatory role of ATP synthase inhibitory factor 1 (ATPIF1) in Wallerian degeneration and peripheral nerve regeneration
Source: Exploration (Beijing). 2024 Mar 19;4(6):20230098. doi: 10.1002/EXP.20230098 (PMC11655313; doi:10.1002/EXP.20230098)
Supplement: Supplementary file 1 — Supporting Information [file EXP2-4-20230098-s004.docx]

Supporting Information

**Decoding the Regulatory Role of ATPase Synthase Inhibitory Factor 1 (ATPIF1) in Wallerian Degeneration and Peripheral Nerve Regeneration**

*Yun Qian* ^1,2#*^, *Zhiwen Yan* ^1,2#^, *Tianbao Ye*^3#^, *Victor Shahin*^4^, *Jia Jiang*^1,2*^, *Cunyi Fan*^1,2*^

1 Department of Orthopedics, Shanghai Sixth People's Hospital Affiliated to Shanghai Jiao Tong University School of Medicine, Shanghai 200233, P. R. China

2 Shanghai Engineering Research Center for Orthopaedic Material Innovation and Tissue Regeneration, Shanghai 200233, P. R. China

3 Department of Cardiology, Shanghai Sixth People's Hospital Affiliated to Shanghai Jiao Tong University School of Medicine, Shanghai 200233, P. R. China

4 Institute of Physiology II, University of Münster, Münster 48149, Germany

#These authors contributed equally to this work.

*Corresponding to:

cyfan@sjtu.edu.cn (Cunyi Fan)

jessicajj19@sjtu.edu.cn (Jia Jiang)

[sakio@sjtu.edu.cn](mailto:sakio@sjtu.edu.cn); [lollipopcloudland@foxmail.com](mailto:lollipopcloudland@foxmail.com) (Yun Qian)

ORCID

Yun Qian: 0000-0003-1600-5693

Zhiwen Yan: 0000-0003-4038-0012

Tianbao Ye: 0000-0002-6884-9612

Victor Shahin: 0000-0003-2710-6473

Jia Jiang: 0009-0002-7616-2498

Cunyi Fan: 0000-0002-7854-5233

**Supplementary Figure 1**


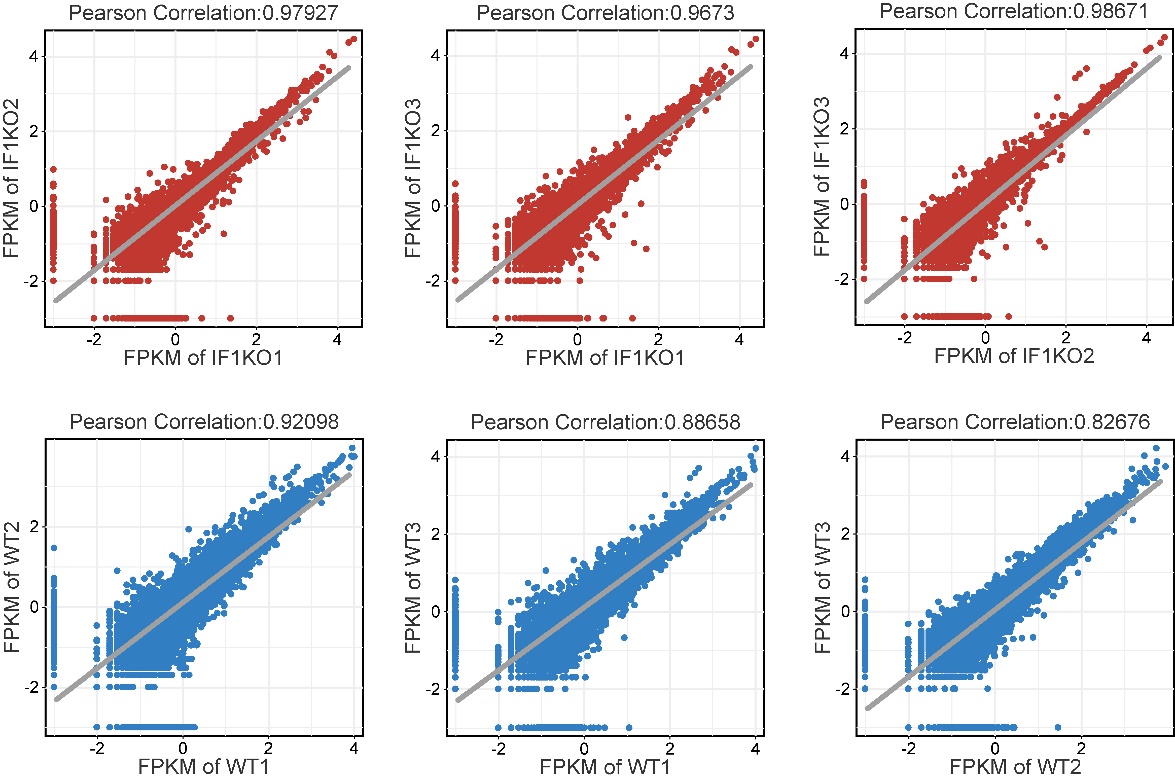


**Figure S1：Pearson’s correlation of the RNA sequencing samples.** FPKM: Fragments Per Kilobase of transcript per Million mapped reads.


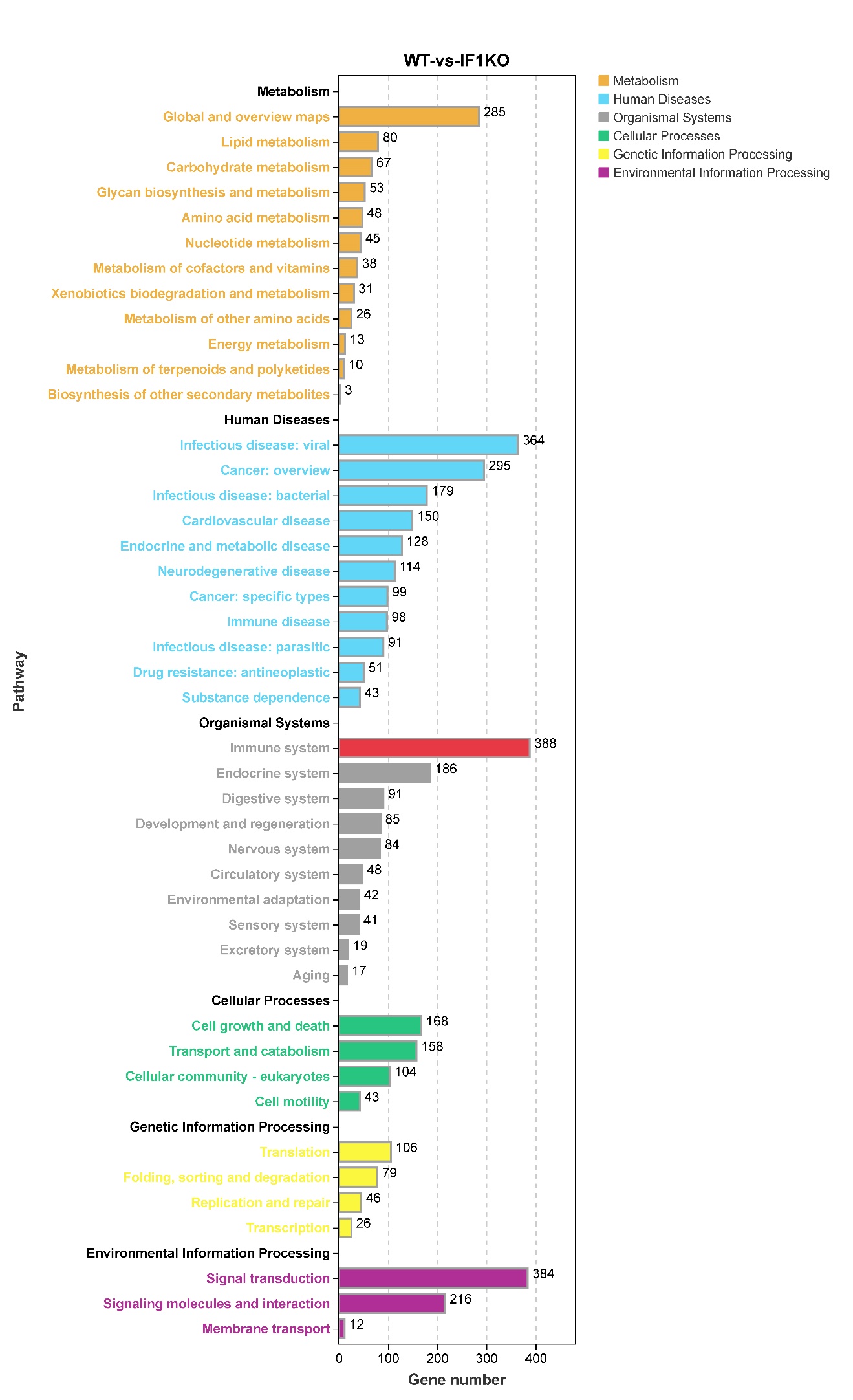


**Figure S2：KEGG enrichment results of the DEGs.** Note the red bar, symbolizing the "Immune System," attains the top position within the "Organismal Systems" category.
